# Physicochemical Characteristics of Chitosan Extracted from *Pleurotus ostreatus* and Its Anticancer Activity Against the MDA-MB-231 Breast Cancer Cell Line

**DOI:** 10.3390/polym17091228

**Published:** 2025-04-30

**Authors:** Adil Aldhahrani

**Affiliations:** Clinical Laboratory Sciences Department, Turabah University College, Taif University, Taif 21995, Saudi Arabia; a.ahdhahrani@tu.edu.sa

**Keywords:** breast cancer, chitosan, anticancer, *Pleurotus ostreatus*

## Abstract

One of the main causes of death worldwide is cancer, a disorder in which a solid or liquid mass of cells known as a tumor develops when one or more cells lose the capacity to regulate their development. This study aims to assess the potential of chitosan as an anticancer agent in place of standard therapy regimens that have some degree of unselective cytotoxicity. PCR was performed for the RNA extraction of Caspase-3 and β-actin genes, and Cq values and quantification curves for each gene were recorded. Additionally, SRB and FITC apoptosis investigations were used to assess the effectiveness of chitosan powder’s anticancer activity against breast cancer cells.

## 1. Introduction

Cancer is a multifaceted disease with very complicated genesis and course. The rise of multidrug resistance and relapse is one of the main issues with anticancer medication development. The DNA of the cell is the direct target of traditional chemotherapeutic medications, but mutations allow the cell to become resistant. Recent advances in the availability of anticancer medications include the creation and subsequent development of novel anticancer small-molecule therapies, as well as molecular-targeted therapy, which targets proteins that exhibit aberrant expression within cancer cells. Numerous intriguing therapeutic targets have been discovered in recent years that could be successfully used to treat cancer. Drug resistance and adverse effects on healthy tissues and cells have frequently hindered the efficacy of targeted chemotherapy, despite its success in treating some cancers. Many individuals who would benefit from targeted chemotherapies are unable to obtain them due to their high cost [1]. In women, non-melanoma skin cancer is the most common cancer diagnosed, followed by breast cancer. Among women worldwide, it is the leading cause of cancer-related mortality. The second leading cause of cancer-related fatalities among women, behind lung cancer, is breast cancer, which affects almost 316,000 people annually in the US [2,3]. A multidisciplinary team of experts in medical, surgical, and radiation oncology is needed to treat breast cancer. Primary care physicians are frequently the first people patients with a new breast cancer diagnosis contact because the majority of breast cancers are detected by mammography or physical examination [4]. *Pleurotus* is a genus of edible mushrooms that produces a considerable number of unique mycochemicals. Due to its short life cycle, high mineral content, therapeutic qualities, repeatability in recycling specific industrial and agricultural wastes, and cheap resource and technological requirements, a number of *Pleurotus* species are now grown commercially [5]. *P. ostreatus*, *P. florida*, *P. flabellatus*, and others belong to the genus *Pleurotus*; some of these are particularly noteworthy because of their high nutritional content and therapeutic significance [6,7]. There are approximately 100 distinct bioactive chemicals in the fruiting body of *P. ostreatus*, and these are mostly believed to be a possible new source of dietary fiber. The fungal cell wall, on the other hand, is rich in non-starch polysaccharides, of which β-glucan is one of the major intriguing functional components, as well as phenolic compounds like protocatechuic acid, tocopherols such as α-tocopherol and γ-tocopherol, homogentisic acid, ascorbic acid, naringin, rutin, myricetin, chrysin, and β-carotene, with each having an exceptional medicinal effect of its own [8,9]. Chitosan is derived from chitin, a naturally occurring biopolymer that is most prevalent in insect cuticles, fungal cell walls, mollusk shells, and crustacean exoskeletons [10]. Chitosan can be chemically modified to produce a number of derivatives with enhanced solubility and several uses [11]. It has been demonstrated that this derivatization of chitosan caused by amino groups and acetamido residues results in compounds with increased solubility and biological activity [12].

This research aims to identify chitosan’s potential as an anticancer agent, which will help to develop safer protocols and less unselective cytotoxic drugs.

## 2. Materials and Methods

### 2.1. Cultivation of Pleurotus ostreatus

A *Pleurotus ostreatus* strain culture with GenBank accession number JN126342 was obtained from the Egyptian Microbial Culture Collection and used to produce chitosan. The active mycelia were collected from a freshly prepared PDA agar plate culture after 7 days of incubation at 25 °C on potato dextrose agar (PDA) with occasional transfers onto a fresh PDA medium. The pre-inoculums were made as follows: 50 mLof MGYP medium (meat extract 2%, peptone 0.5%, yeast extract 0.3%, and glucose 1.5%). Without any disruption, the Erlenmeyer flask that had been inoculated was incubated at 28 °C for 15 days. Following a 15-day incubation period, the *Pleurotus* ostreatus fungal mycelium was isolated and filtered to produce a consistent weight for the extraction of chitosan and chitin. The mycelium mat was dried at 60 °C after being rinsed with sterile distilled water until a clear filtrate was produced using Whatman No. 1 filter paper (MRS Scientific Ltd., Wickford, UK).

### 2.2. Production of Chitosan and Extraction of Chitin

Using the alkalization process, chitosan was obtained from the chitin extract [13,14]. After being dried for two days at 60 °C in an oven, the fresh fungal mycelium with different biomasses, shown in Table 1, was ground into a powder. After being treated with a 1 N NaOH solution, the powder was stored at 100 °C for three hours. The slurry was filtered in order to collect the alkali-insoluble materials (AIMs). The AIMs were dried for four days at 40 °C in an oven. A water bath heated to 100 °C was then used to dilute the dried AIMs in 2% acetic acid (Thermo Scientific Chemicals, Waltham, MA, USA, 99.5% pure) for five hours. The supernatant was then poured into a beaker after the sample solution was centrifuged for ten minutes at 6000 rpm. Centrifugation at 6000 rpm for 5 min was used to recover the precipitation after adjusting the pH to 12.0 using a 2 N NaOH solution. Following the same centrifugation parameters, the precipitate was rinsed 4 or 5 rounds with distilled water before being dried at 45 °C in an oven.

### 2.3. Characterization of Pleurotus ostreatus

The strain was characterized through chemical composition analysis to obtain moisture, fats, protein, ash, and carbohydrate contents; the procedures utilized are described in detail in the next sections.

#### 2.3.1. Chemical Composition of Pleurotus ostreatus Biomass

The chemical composition of *Pleurotus ostreatus* was determined according to the official methods of analysis mentioned in AOAC INTERNATIONAL [15]. Briefly, a hot-air oven was utilized to determine moisture content, protein content was determined according to the Kjeldahl method, the acid hydrolysis method was used to determine total fat, and the gravimetric method was used to determine ash. Three replicates were used to assess the percentages of moisture, protein, fat, and ash. The amount of carbs was then obtained by calculating the difference between the chemical composition results using the following equation: moisture + protein + fat + ash = 100 − (fresh weight); this equals total carbs.

#### 2.3.2. Moisture and Ash Content Determination in Biomass

Following a 0.5 g mass weighing of the chitosan for each sample, the moisture content of the samples was measured after being subjected to the moisture analyzer. For every sample, the values that were obtained were expressed as percentages. Weighed empty crucibles were labeled as Ma to indicate the amount of ash. The masses were expressed in Mb, and each sample was weighed to a mass of 0.1 g. The samples were placed in a crucible and exposed to an open flame until the chitosan ceased producing white fumes and turned black. A furnace was then used to burn the crucibles for 12 h at 550 °C. After weighing the samples again, the crucibles were assigned the number Mc. The following formula was used to determine each sample’s ash content: ash content (%) = 100 × (Mc − Ma/Mb).

### 2.4. Characterization of Chitosan

Chitosan’s inherent viscosity, molecular weight, degree of deacetylation, and nitrogen concentration were measured according to the methods mentioned below.

#### 2.4.1. Determination of Nitrogen Content

According to ISO 16948:2015, the nitrogen (N) concentration was determined using the Vario MACRO CHNS from Elementar Analysensysteme GmbH (Langenselbold, Germany) [16]. After the combustion tube was heated to 1150 °C, 30 mg of homogenized samples was placed on the carousel of an automatic sample feeder and covered with tin foil.

#### 2.4.2. Determination of Average Viscosity Molecular Weight

The molecular weight of chitosan was determined using the Ostwald viscometer through the comparison of the viscosity of a known solvent (0.3 M acetic acid + 0.2 M sodium acetate) with the viscosity of an unknown liquid (sample solution). Two liquids of the same volume were compared for flow times (tsolvent = solvent flow time and t sample = sample flow time) in order to measure the viscosity of the liquid using the same viscometer. Furthermore, the values of average runtime, specific viscosity (•spe), and reduced viscosity (•red) were calculated using the measured flow times. Moreover, the graph was created by plotting the concentration of chitosan (x-axis) against reduced viscosity (y-axis). The intrinsic viscosity [•], which is equivalent to the Y-intercept, was then determined by creating an extrapolation plot using a trend line showing a decrease in the viscosity relative to the concentration of chitosan. The following formula was used to determine the sample’s intrinsic viscosity: (Runtime A + Runtime B + Runtime C)/3 = the average runtime.

Specific viscosity (•spe) = (Sample runtime − Solvent runtime)/Solvent runtime

Reduced viscosity (•red) = Specific viscosity/Sample concentration

Intrinsic viscosity = Y − intercept of the plot

The Mark–Houwink equation was used to calculate the molecular weight [17].

η = KM^a^;
η = Intrinsic viscosity;
K = Constant;
M = Relative molecular mass average;
a = Solvent’s relative constant.

#### 2.4.3. Gas Chromatography–Mass Spectrometry (GC-MS) Analysis

Analysis was carried out using a GC-TSQ mass spectrometer (Thermo Scientific, Austin, TX, USA) with a direct capillary column TG–5MS (30 m × 0.25 mm × 0.25 µm film thickness). The column oven temperature was initially held at 60 °C and then increased by 5 °C/min to 250 °C, then increased to 300 °C with a heating rate of 3 °C/min after being held for 2 min. The injector temperature was kept at 270 °C. Helium was used as a carrier gas at a constant flow rate of 1 mL/min. The solvent delay was 4 min, and 100 µL of derivatization reagent (80 µLBFSTA + 20 µLTMCS) was added to the dried sample that was incubated at 65 °C for one hour before injection. Diluted samples of 1 µL in volume were injected automatically using Autosampler AS3000 (Thermo Fisher Scientific, Waltham, MA, USA) coupled with GC in the split mode. EI mass spectra were collected at 70 eV ionization voltages over the range of *m*/*z* 50–650 in full scan mode. The ion source and transfer line were set at 200 °C and 280 °C, respectively. The components were identified by comparing their mass spectra with those of WILEY 09 and NIST14 mass spectral database [18,19].

#### 2.4.4. Water-Binding Capacity Determination (WBC)

The Knorr method [20] was used to measure the WBC of chitosan. By weighing a tube that contained 0.5 g of chitosan, WBC was determined. After adding 10 milliliters of water (Paul Muller Company, Springfield, MO, USA, 100% pure) to help disperse the material, the mixture was vortexed for one minute. The contents were allowed to stand at room temperature for 30 min with 5 s of sporadic shaking every 10 min and were then centrifuged for 25 min at 3000 rpm. Following the decantation of the supernatant, the tube was weighed once more. The bound water (g) was computed by subtracting the weight of the centrifuge tube containing chitosan and water from the weight of the tube containing only chitosan. The formula used to determine WBC is as follows: WBC (%) = [Water bound (g)/Initial sample weight (g)] × 100.

#### 2.4.5. Fat-Binding Capacity Determination (FBC)

The Knorr method [20] was used to measure the FBC of chitosan [20]. The same procedure outlined in WBC was performed to determine FBC, and soybean oil (Vigon International, an Azelis Company, East Stroudsburg, PA, USA, 100% pure) was substituted for water. The formula used to compute FBC is as follows: FBC (%) = [Fat bound (g)/Initial sample weight (g)] × 100.

#### 2.4.6. Fourier Transform Infrared Spectroscopy (FTIR)

Infrared spectroscopy utilizing the KBr pellet method in FTIR (Shimadzu FTIR-8200, Kyoto, Japan) was used to confirm the structure of the isolated mycelia chitin and chitosan. With a resolution of 4 cm^−1^, FTIR spectra were acquired in the absorbance mode for 16 scans in the middle infrared (4000 cm^−1^ to 400 cm^−1^) at ambient temperature. The mycelial chitosan samples were prepared by grinding dry mycelial chitosan powder with powdered KBr (ACG-EGYPT, Nasr City, Egypt, 99.5% pure) in a 1:5 ratio (sample–KBr). The KBr pellet was then compressed and subjected to FTIR analysis.

#### 2.4.7. Solubility Determination

The following formula was applied to ascertain each sample’s solubility: solubility (%) = 100 × (Ma − Mc/Ma). For each of the samples, the chitosan was weighed to a mass of 0.1 g; the masses are represented by the symbol Ma. After that, the samples were combined and dissolved in a 30 mL solution of 1% ethanoic acid. After stirring for 30 min, the undissolved residues were filtered out and dried in an oven.

#### 2.4.8. Degree of Deacetylation

Using three distinct absorbance ratios (R = A1655/A2870, A1655/A3450, and A1320/A1420) in a comparable equation, the absorbance wavenumbers derived from FTIR spectroscopy study were used to calculate the chitosan’s deacetylation levels [21]: Deacetylation degree (%) = 100% − (R − 0.03822/0.03133).

### 2.5. Real Time PCR for Caspase-3 and β-Actin Genes in Chitosan

An RNeasy Mini kit (Cat. No. 74104, QIAGEN, Hilden, Germany) was applied to the samples in compliance with the manufacturer’s directions to extract total RNA. An A260/280 ratio Plate reader FLUOstar Omega (BMG LABTECH company, Offenburg, Germany) Spectrophotometer was used to measure the RNA’s purity and concentration. Using a total volume of 20 µL, which included 2 µL DNA (ADVENT CHEMBIO PVT. LTD., Maharashtra, India, 100% pure), 2 µL (0.2–0.5 µM) forward and reverse primers, 10 µL master mix, and 6 µL nuclease-free water, the SYBR Green qPCR master mix (TransGen Biotech company, Haidian District, Beijing, China) and the Bio-Rad platform (Bio-Rad CFX OPUS 96, BIO RAD company, Dubai, United Arab Emirates) were used to determine the levels of gene expression.

### 2.6. Sulforhodamine B (SRB) Viability Test in Cancer Cells

#### 2.6.1. Cell Culture

Nawah Scientific Inc. provided the MDA-MB-231: Breast Cancer Cell Line (Mokatam, Cairo, Egypt). At 37 °C in a humidified, 5% (*v*/*v*) CO_2_ environment, the cells were cultured in DMEM media formulated with 100 units/mL penicillin (OXOID, Basingstoke, Hampshire, UK, 100% pure), 100 mg/mL streptomycin (OXOID, UK, 100% pure), and 10% heat-inactivated fetal bovine serum (Genetics company for Biotechnology, Amman, Jordan, 100% pure).

#### 2.6.2. Cytotoxicity Assay

The SRB test was used to evaluate the viability of the cells after they were cultured in DMEM media along with 100 units/mL penicillin. In 96-well plates, aliquots of 100 μL cell suspension (5 × 10^3^ cells) were incubated for 24 h in full media. The cells were treated with another aliquot of 100 μL media that included drugs in varying quantities. After the drug treatment, the cells were fixed with 150 μL of 10% TCA (Ataman chemicals, Istanbul, Turkey, 80% pure) added to the media, and they were then incubated at 4 °C for an hour. After removing the TCA solution, distilled water was used to wash the cells five times. After adding 70 μL aliquots of SRB solution (0.4% *w*/*v*), the mixture was allowed to sit at room temperature for 10 min in a dark environment. After three rounds of washing with 1% acetic acid, the plates were set to air dry overnight. Following the dissolution of the protein-bound SRB stain with 150 μL of TRIS (10 mM), the absorbance at 540 nm was measured using an Infinite F50 microplate reader (TECAN, Männedorf, Switzerland) [22,23].

### 2.7. FITC Apoptosis Assay for Cancer Cells

#### 2.7.1. Cell Culture

Nawah Scientific Inc. provided the MDA-MB-231: Breast Cancer Cell Line (Mokatam, Cairo, Egypt). A 10-percent heat-inactivated fetal bovine serum, 100 units/mL penicillin, and 100 mg/mL streptomycin were added to DMEM media, which were used for culturing the cells at 37 °C in a humidified environment with 5% (*v*/*v*) CO_2_.

#### 2.7.2. Flow Cytometry Assay

An Annexin V-FITC apoptosis detection kit (Abcam Inc., Cambridge Science Park, Cambridge, UK) in combination with flow cytometry using two fluorescent channels was employed to identify apoptosis and necrosis cell populations. Following a specified duration spent in treatment with the test chemicals, 105 cells underwent the trypsinization process and then rinsed twice with ice-cold PBS (pH 7.4). Then, in agreement with the manufacturer’s instructions, the cells were incubated for 30 min at room temperature in the dark with 0.5 mL of the Annexin V-FITC/PI solution. AnACEA Novocyte™ flow cytometer (ACEA Biosciences Inc., San Diego, CA, USA) was used to inject the cells after staining. The FL1 and FL2 signal detectors were used to detect FITC and PI fluorescent signals, respectively (λex/em 488/530 nm for FITC and λex/em 535/617 nm for PI). Using ACEA NovoExpress™ software (version 1.6.2) (ACEA Biosciences Inc., San Diego, CA, USA), 12,000 incidents were collected for each sample, and positive FITC and/or PI cells were counted using quadrant analysis [24,25,26,27].

## 3. Results

### 3.1. Biomass of Pleurotus ostreatus and Yield of Chitin and Chitosan

Following 15 days of incubation at 28 °C in an Erlenmeyer flask containing *Pleurotus* ostreatus mycelia, the mat was washed and dried. Throughout the incubation period, the dry weight of the mat, the yield of extracted chitin, and the resultant chitosan were continuously measured (Table 1).

### 3.2. Characterization of Pleurotus ostreatus Biomass

“Official Methods of Analysis of AOAC International” were followed in the analysis of *Pleurotus* ostreatus’ chemical composition, and the findings were documented (Table 2).

### 3.3. Characterization of Produced Chitosan

#### 3.3.1. Chitosan’s Molecular Weight, Intrinsic Viscosity, and Degree of Deacetylation

Following the examination of chitosan composition and structure, each of the characterized parameters was measured, and the information obtained was documented (Table 3).

#### 3.3.2. GC-MS Analysis

**Figure 1:**
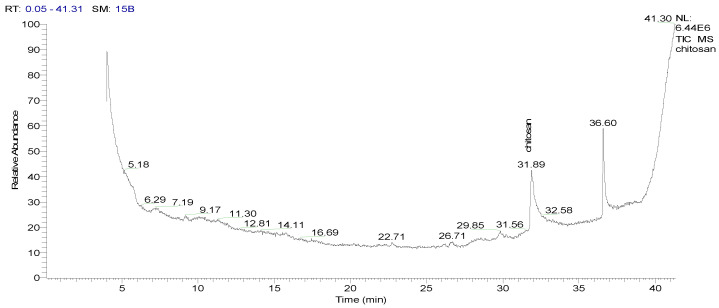
GSMS chromatogram showing the abundance (*y*-axis) of chitosan at a retention time (*x*-axis) of 31.89 min.

**Figure 2:**
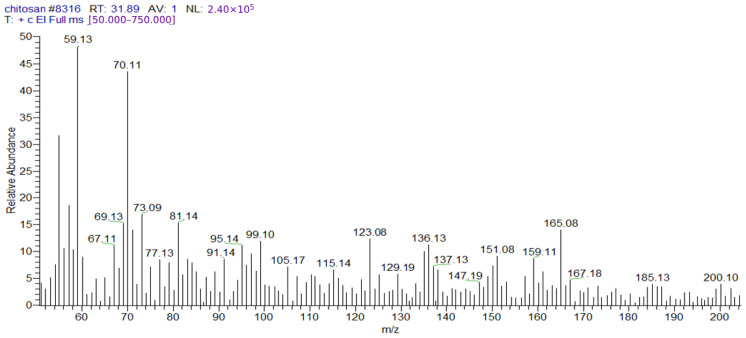
Mass spectrum of chitosan showing the reference base peak at *m*/*z* = 59; peaks with relative intensities smaller than 5% of the reference peak were excluded to avoid background contamination ions.

After injecting the sample into the GC-TSQ mass spectrometer, the sample was separated using gas chromatography features, and then the neutral molecules were eluted through a heated transfer line into the mass spectrometer. Moreover, the ionization of these molecules was achieved using electron ionization, resulting in unstable molecular ions, which can lose their excess energy via fragmentation. Furthermore, the produced fragments of different masses were separated using the mass analyzer; after that, they were subjected to ion detection, the signals of all the fragments were recorded (Table 4), and the chromatograms were generated (Figure 1 and Figure 2).

#### 3.3.3. Solubility of Chitosan

After standing for 24 h at room temperature, the degree of solubility was tested at a concentration of 10 mg/mL in each solvent. The solvents in which solubility was assessed were H_2_O_2_, as well as various concentrations of CH_3_COOH and NaOH (Table 5).

#### 3.3.4. Capacity of Chitosan to Bind Fat and Water

The water-binding capacity (WBC) and fat-binding capacity (FBC) were calculated using essentially the same equation except that the parameter indicating the fat bound in grams was used in the equation to measure the fat-binding capacity, whereas this was replaced with the weight of water bound for calculating the water-binding capacity; the values represent these capacities are listed in Table 6.

#### 3.3.5. Fourier Transform Infrared (FTIR) Spectroscopy

**Figure 3:**
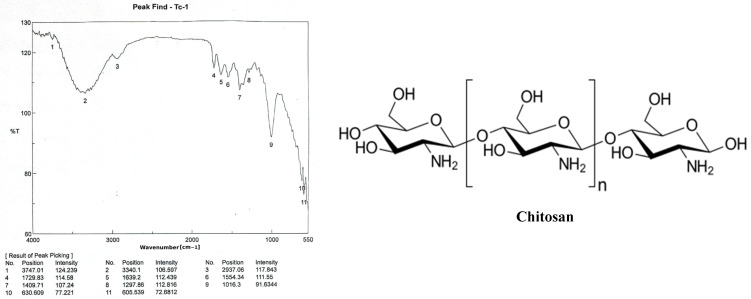
Chitosan [poly-(β-1/4)-2-amino-2-deoxy-D-glucopyranose] infrared spectrum with band positions and intensities.

The absorption band at 2937 cm^−1^ is attributed to C-H symmetric stretching, while the strong band in the range 3340 cm^−1^ in the chitosan infrared spectrum is associated with OH stretching. These bands represent typical polysaccharide properties (Figure 3).

### 3.4. PCR for the RNA Extraction of Selected Chitosan Genes

**Figure 4:**
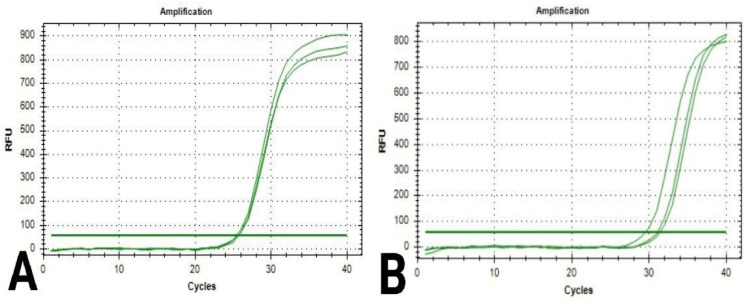
Quantification curves for Caspase-3 gene in breast cancer cells: (**A**) cells in the control sample; (**B**) cells in the chitosan sample.

**Figure 5:**
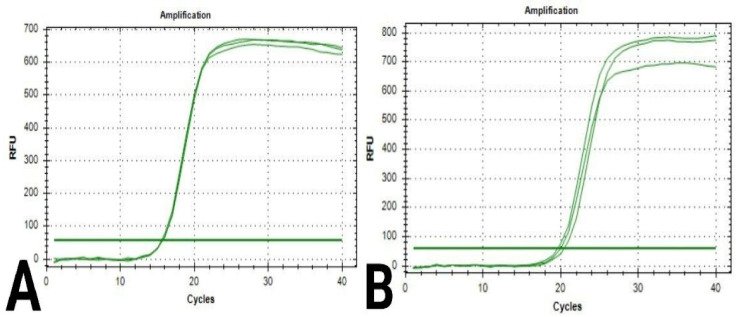
Quantification curves for β-actin gene in breast cancer cells: (**A**) cells in the control sample; (**B**) cells in the chitosan sample.

During the analysis of quantification curves (Figure 4 and Figure 5), the cycle number at which the fluorescence first reaches above the threshold level for both is known as the quantification cycle, or Cq; this value was recorded for Caspase-3 and β-actin genes in breast cancer cells in both chitosan and control samples (Table 7 and Table 8).

### 3.5. SRB Viability Test

**Figure 6:**
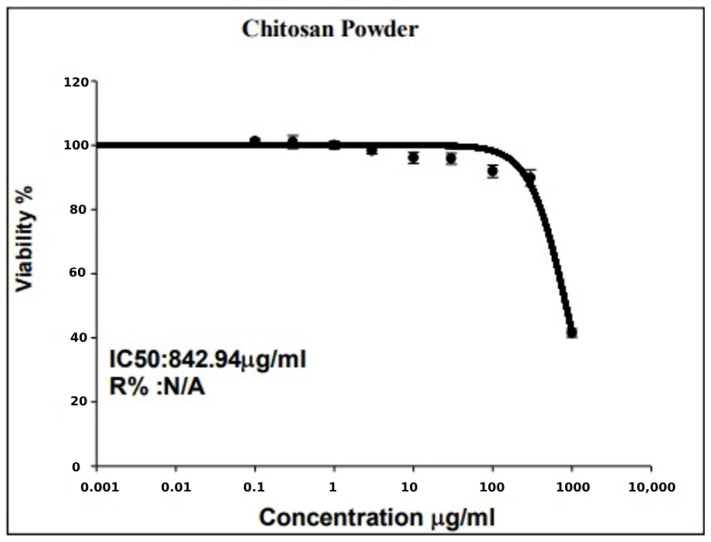
Dose–response curve displays the value of IC50 for chitosan considering the relationship between each concentration of chitosan and the resultant percentage of viable cells.

Subsequent to the incubation of cells with different concentrations of chitosan, at a wavelength of 540 nm, optical density values were measured with a 96-well micro-titer plate reader. The formula used to calculate the percentage of growth inhibition (I%) is I% = (1 [ODt/ODc] × 100%), where ODt and ODc stand for the optical density values of the test and control samples, respectively. To calculate the IC50 value, dose–response curves were plotted, and the percentage of viable cells at each concentration was recorded (Table 9 and Figure 6).

### 3.6. FITC Apoptosis Test

**Figure 7:**
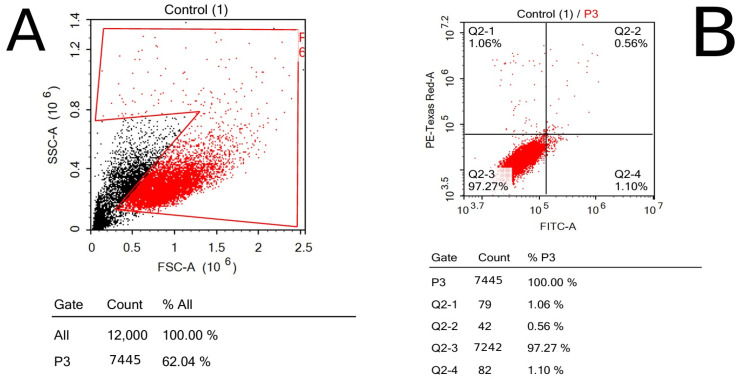
Control sample’s flow cytometry using dot plotting; the results show the total fluorescence of the cell population: (**A**) forward and side scatter of cells representing the size and complexity of cells; (**B**) quadrant analysis of cells shows the number of cells in each phase of apoptosis.

**Figure 8:**
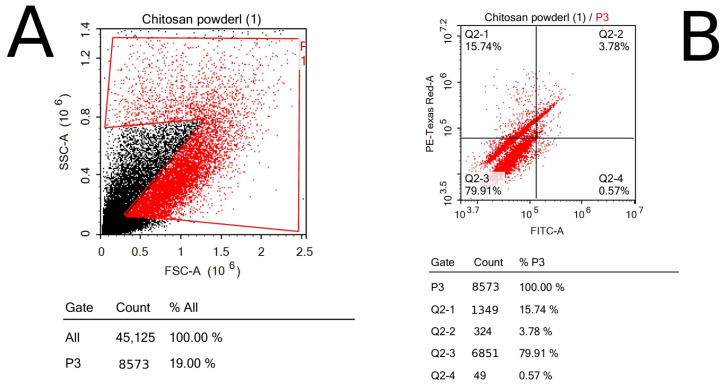
Chitosan sample’s flow cytometry using dot plotting; the results show the total fluorescence of the cell population: (**A**) forward and side scatter of cells representing the size and complexity of cells; (**B**) quadrant analysis of cells shows the number of cells in each phase of apoptosis.

To evaluate the viability of the cells that were exposed to chitosan powder in comparison with the control sample, ACEA NovoExpress™ software (version 1.6.2) (ACEA Biosciences Inc., San Diego, CA, USA) was used to quantify and analyze the PI cells through quadrant analysis; thus, all apoptotic phases of breast cancer cells, including early (Q2-4) and late (Q2-3) apoptotic phases and necrosis phase (Q2-1), were detected, in addition to the normal state of cells (Q2-2) (Figure 7 and Figure 8).

## 4. Discussion

Chitosan is a cationic polymer that is primarily produced by N-deacetylation chitin. It is a copolymer of N-acetyl glucosamine and glucosamine unit. Because of its biodegradability and biocompatibility, it has been widely used in biological and biomedical applications [28]. As chitosan is nontoxic, biocompatible, biodegradable, and adsorptive, it can be used for a variety of purposes. In several research studies, chitosan has been found to have anticancer properties with low toxicity to noncancer cells. Its capacity to be effective against various cancer cell types is mainly dependent on DDA and molecular weight [29,30,31,32]. In this research, chitosan was obtained from *Pleurotus ostreatus* fungi, and both Caspase-3 and β-actin were characterized through the analysis of chemical compositions and characteristics. The chemical composition analysis of *Pleurotus ostreatus* was performed according to the standard methods indicated in several previously published studies [33,34,35]. The solubility of chitosan in different solvents, such as hydrogen peroxide, sodium hydroxide, and acetic acid, was determined. Moreover, the molecular mass analysis of chitosan was carried out by means of both intrinsic viscosity and GC-MS spectrometry; in addition to that, water- and fat-binding capacities of chitosan were identified [16,17,18,19]. The IR spectrum of chitosan was acquired using the FTIR technique; OH stretching is represented by the strong band in the spectrum at 3340 cm^−1^, whereas the absorption band at 2937 cm^−1^ is attributed to C-H symmetric and asymmetric stretching, similar to the bands detected in IR spectrums in other similar previous studies [36]. The values obtained from assessing both fat- and water-binding capacities were almost typical and similar to what has been reported in other scientific publications [37,38]. Following [39], PCR was performed for Caspase-3 and β-actin genes in breast cancer cells exposed to chitosan to obtain the Cq values and amplification curves for both genes. The viability of breast cancer cells subjected to chitosan was investigated through SRB and FITC apoptosis examinations, and the results showed the ability of chitosan against cancer cells, in agreement with many previous studies; in addition to that, the quadrant analysis of FITC showed a considerable number of cells in both late and early apoptotic phases [40,41].

## 5. Conclusions

Briefly, and from the authors’ point of view, relying on safety features offered by using chitosan, which include nontoxicity, biocompatibility, and biodegradability, and the efficacy demonstrated through the results obtained from the assays performed in our study, such as SRB viability assay and FITC apoptosis assay, chitosan holds promise as an anticancer agent, which can be confirmed by performing more studies in the future.

## Figures and Tables

**Table 1:** Yield of biomass, chitin, and chitosan through the incubation period of 15 days.

Day	Yield of Biomass (g/L Dry wt)	Chitin Yield (mg/L)	Chitosan Yield (mg/L)
3	1.23	18.5	0.084
5	3.84	56	0.342
8	7.56	182	0.653
12	11.83	298	173.8
14	10.0	214	83.12

**Table 2:** The percentage of each chemical component measured using chemical analysis.

Components	Percentage %
Moisture content (%)	92
Fats	0.3
Protein	3.2
Ash content (%)	0.8
Carbohydrates	3.6

**Table 3:** The characterization parameters of chitosan.

Parameter	Value
Intrinsic Viscosity (dL/g)	765.0
Molecular weight	1.6 × 10^5^ Da
Degree of deacetylation (DD%)	80.0%
Nitrogen content (N%)	0.54% dry weight of biomass

**Table 4:** Mass-to-charge ratio, absolute intensity, and relative intensity of all the fragment ions detected.

Mass-to-Charge Ratio (*m*/*z*)	Absolute Intensity	Relative Intensity
55.08	76,135.4	31.67
56.12	25,497.2	10.61
59.18	141,858.2	59.01
58.14	24,835.2	10.33
60.19	21,510	8.95
67.11	26,771.6	11.14
69.13	36,599.6	15.22
70.11	240,394.6	100
71.12	33,801.5	14.06
73.09	40,699.6	16.93
81.14	36,989.8	15.39
95.14	26,915	11.2
97.13	23,026.7	9.58
99.1	28,645.9	11.92
123.08	29,649	12.33
135.18	24,164.3	10.05
136.13	26,941.6	11.21
151.08	21,880.9	9.1
159.11	21,013.8	8.74
165.08	33,782.6	14.05

**Table 5:** Percentage of solubility of chitosan in a variety of solvents.

Treatments	Solubility Case	Solubility %
H_2_O_2_	Insoluble	0.00
CH_3_COOH (0.1%)	Swelling	21.6 ± 1.5
CH_3_COOH (0.5%)	Soluble	95.8 ± 0.72
CH_3_COOH (1%)	Soluble	100 ± 0.00
NaOH (1%)	Insoluble	0.00

**Table 6:** Water-binding (WB) and fat-binding (FB) capacities of chitosan.

Replicates	Initial Weight (g)	Chitosan’s Water-Binding (WB) Capacity			Chitosan’s Capacity to Fat Binding (FB)		
		Weight of Water Bound	WBC (%)	Ave. WBC (%)	Weight of Fat Bound	FBC (%)	Ave. FBC (%)
1	0.5	2.62	524	526.6 ± 8.3	1.78	365	363 ±4.3
2	0.5	2.68	536		1.83	366	
3	0.5	2.60	520		1.79	358	

**Table 7:** Cq for Caspase-3 gene in breast cancer cells in chitosan and control samples.

Sample	Cq for Caspase-3		
	Result 1	Result 2	Result 3
Control	25.66679	25.4567	25.73934
Chitosan	31.37618	29.50236	31.10158

**Table 8:** Cq for β-actin gene in breast cancer cells in chitosan and control samples.

Sample	Cq for β-Actin		
	Reading 1	Reading 2	Reading 3
Control	15.56439	15.68983	15.74965
Chitosan	20.39383	19.97067	19.55612

**Table 9:** Different treatment concentrations of chitosan during incubation and percentages of viability.

Chitosan	Raw Data			Blank Corrected Data			Viability%				
Conc.	1	2	3	1	2	3	1	2	3	Mean	STD
C	2.2304	2.207	2.221	2.195	2.1716	2.1856	100	100	100	100	0
0.1	2.2223	2.2512	2.2649	2.1869	2.2158	2.2295	100.13	101.453	102.08	101.221	0.813004658
0.3	2.2223	2.1993	2.3033	2.1869	2.1639	2.2679	100.13	99.0766	103.838	101.015	2.042265236
1	2.2442	2.1807	2.2364	2.2088	2.1453	2.201	101.132	98.225	100.775	100.044	1.294630271
3	2.1884	2.155	2.1998	2.153	2.1196	2.1644	98.5776	97.0483	99.0995	98.2418	0.870413289
10	2.1022	2.188	2.1142	2.0668	2.1526	2.0788	94.6308	98.5593	95.1802	96.1234	1.736931189
Blank	0.0358	0.035	0.0354	Blank average		0.0354	Control average		2.18407		

## Data Availability

The study’s original contributions are highlighted in the article’s Methods and Materials section; additional information is provided by contacting the study’s corresponding author.
